# Infantile inflammatory bowel disease in three Syrian infants: a case series

**DOI:** 10.1186/s13256-024-04456-9

**Published:** 2024-03-18

**Authors:** Afif Alshwaiki, Ranim M. H. D. Samir Nakhal, Ali Alakbar Nahle, Hussein Hamdar, Nafiza Martini, Jaber Mahmod

**Affiliations:** 1 Faculty of Medicine, Damascus University, Damascus, Syrian Arab Republic; 2 Stemosis for Scientific Research, Damascus, Syrian Arab Republic

**Keywords:** Inflammatory bowel diseases, Crohn’s disease, Ulcerative colitis, Very early-onset IBD (VEO-IBD), Infants, Case series

## Abstract

**Background:**

Inflammatory bowel diseases, consisting of Crohn’s disease and ulcerative colitis, are chronic bowel relapsing inflammatory disorders. Inflammatory bowel diseases begin rarely in infants. Approximately 25% of patients with inflammatory bowel diseases present before the age of 20 years. Very early-onset inflammatory bowel disease occurs before the age of 6 years; infantile inflammatory bowel diseases occurs before the age of 2 years, and is extremely rare in infants under 1 year of age.

**Case presentation:**

Herein, we report a case series of 7-month-, 11-month-, and 12-month-old Syrian infants that presented with diarrhea, hematochezia, and pale appearance and were finally diagnosed with infantile inflammatory bowel disease and treated.

**Conclusions:**

Early diagnosis and ruling out infantile inflammatory bowel diseases despite its rarity are recommended. Over and above that, new drugs such as vedolizumab, golimumab, and less invasive treatment methods should also be taken into consideration for better response and adequate remission with improved quality of life.

## Introduction

Inflammatory bowel diseases (IBDs), consisting of Crohn’s disease (CD) and ulcerative colitis (UC), are chronic bowel relapsing inflammatory disorders [1]. CD can affect any segment of the gastrointestinal tract (usually, the terminal ileum or the perianal region) with a transmural inflammation with sporadic pattern. On the other hand, UC causes mucosal inflammation and is limited to the colon. The complications such as abscesses, fistulas, and strictures take place in CD more than in UC [2].

IBDs begin most commonly during puberty and young adulthood, and rarely in infants [3, 4]. Approximately 25% of patients with IBDs present before the age of 20 years [5]. The peak onset of IBD among children is in adolescence, with 4% present before age 5 years and 18% before age 10 years [6]. Very early-onset IBD (VEO-IBD) occurs before the age of 6 years; infantile IBD occurs before the age of 2 years and is extremely rare in infants under 1 year of age [7–9]. Genetic, familial, and external environmental factors are implicated [10, 11]. Childhood-onset IBD clinical characteristics usually progress rapidly and more aggressively in comparison with the features of the adult cohort, hence early diagnosis is imperative [8, 12]. Thus, any child with persistent diarrhea, hematochezia, failure to thrive, and/or poor feeding should have IBD considered in the differential diagnosis. The diagnosis of IBD is based on a set of clinical, biochemical, stool, endoscopic, cross-sectional imaging, and histological investigations [13].

We report herein a case series of 7-month-, 11-month-, and 12-month-old infants that presented with diarrhea, hematochezia, and pale appearance and were finally diagnosed with infantile IBD and treated.

## Case presentation

### Case 1

**Fig. 1:**
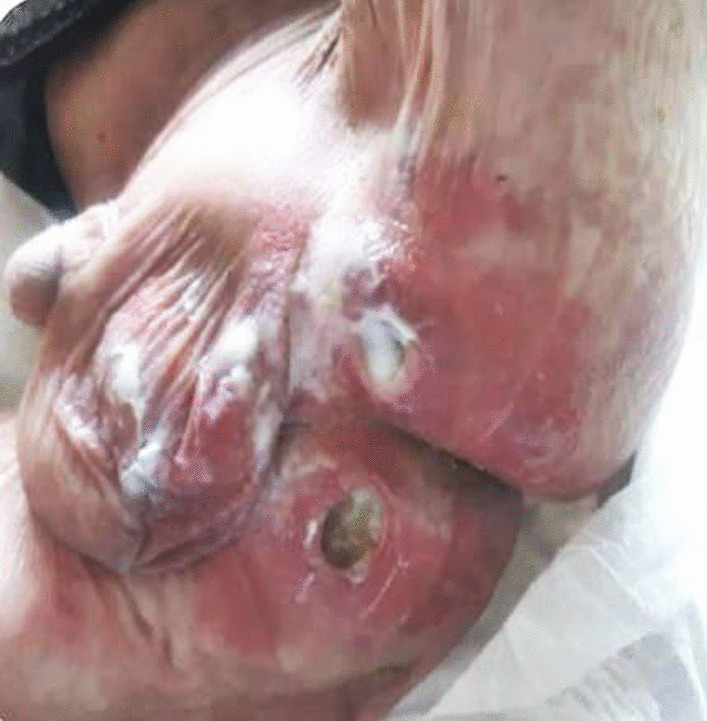
Severe diaper dermatitis with ulcers and perianal material loss

**Fig. 2:**
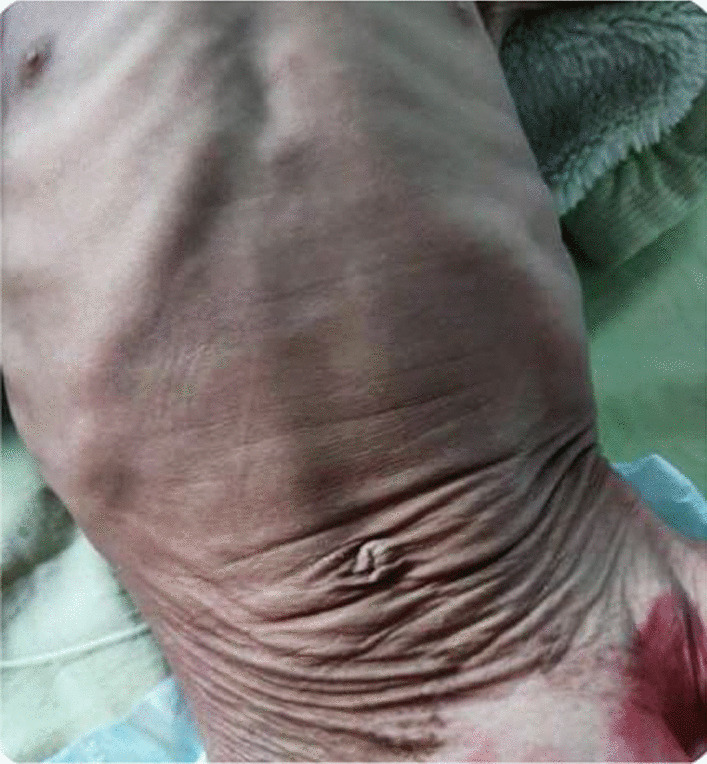
Moderate acute malnutrition (MAM)

**Fig. 3:**
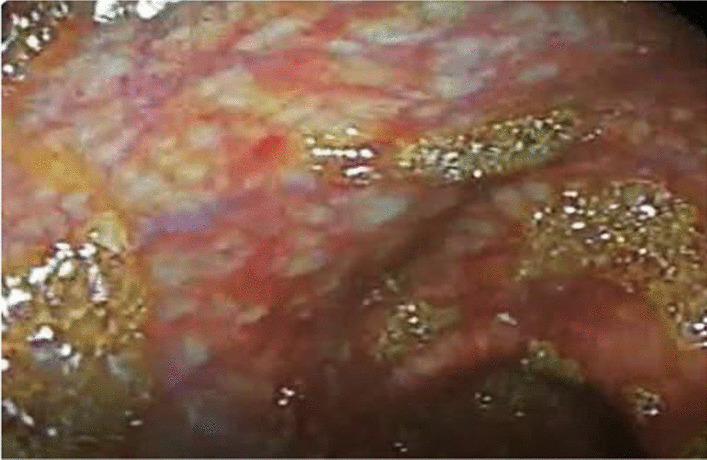
Colonoscopy shown erythematous region containing shallow ulcers in many focal sites of colonic mucosa

This patient was an 11-month-old Syrian male who presented with mild fever (38–38.5 °), diarrhea 1 month ago, and blood in stool with pale appearance 3 days before accepting him into our hospital. Clinical examination showed severe pallor, moderate acute malnutrition (MAM), pitting edema grade II, acute diaper dermatitis with ulcers, and perianal material loss (Figs. 1, 2). The patient had a 43 cm head circumference, 71 cm length, and 7.3 kg weight. He had normal delivery and normal birth weight. The initial laboratory results revealed that the patient had normocytic anemia (Hb 7.4 g/dl, MCV 78 fl), low albumin (2 g/dl), and low total protein (TP) (3.5 g/dl). Both x-ray and abdominal echography showed to be normal. A fecal test showed positive white blood cells in the stool (+4 WBCs), positive red blood cells in the stool (+4 RBCs), and negative fecal culture. Our patient’s fecal calprotectin was measured and was highly elevated (957 mg/kg), and owing to this high level of calprotectin, the patient underwent a colonoscopy (Fig. 3). This showed us an erythematous region containing shallow ulcers in many focal sites of colonic mucosa. During colonoscopy, multiple biopsies were taken and subsequently showed abscess formation in the crypts of the colonic mucosa, leading to a diagnosis of ulcerative colitis. For treatment, we administered oral 5-aminosalicylic acid 50 mg/kg/day for 24 weeks, and metronidazole 15 mg/kg/day in three divided doses for 10 weeks. After 3 months, his hemoglobin was normal and he had gained 0.5 kg in weight. After 6 months, there was no diarrhea and a negative fecal occult blood test.

### Case 2

**Fig. 4:**
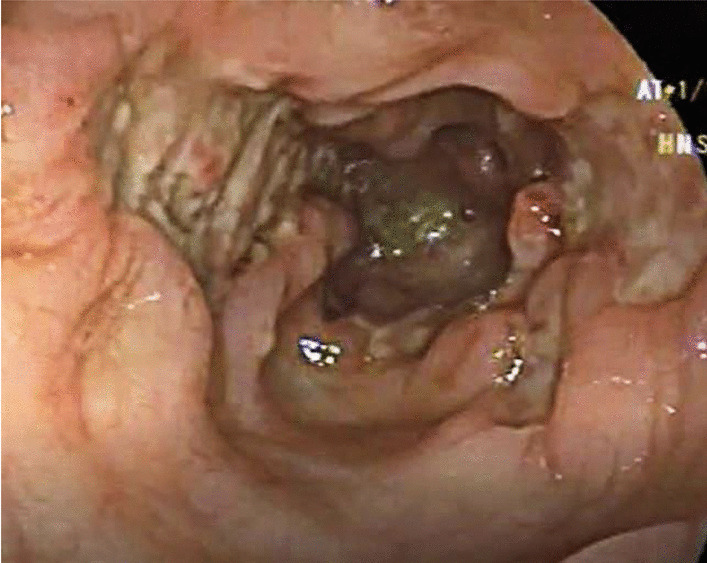
Colonoscopy showing ulcers in the ascending colon

**Fig. 5:**
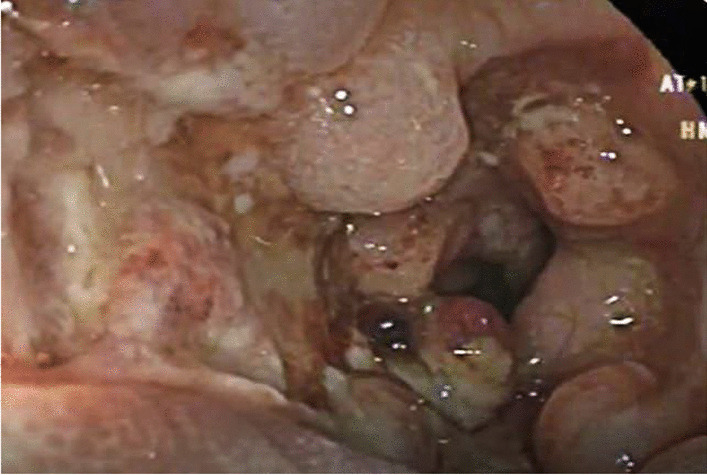
Colonoscopy showing numerous pseudopolyps

**Fig. 6:**
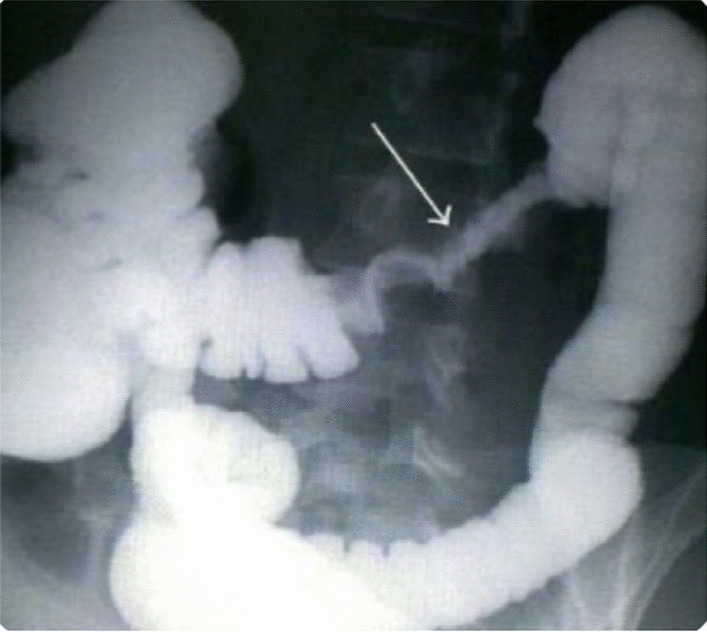
Barium enema demonstrating strictures and narrowing in the transverse colon (arrow)

The next case was a 7-month-old Syrian female who presented pallor along with a history of diarrhea that started 2 months ago and intermittent hematochezia starting from the age of 1 month. We noticed on clinical examination a low-grade pallor, severe acute malnutrition, and severe diaper dermatitis with rash and exfoliation. A laboratory test revealed microcytic anemia (Hb 8.4 g/dl—MCV 69). Cow’s milk allergy test through a skin prick showed to be negative. Moreover, immunoserology was noted to be negative. Fecal calprotectin was on normal limits (54 mg/kg). Abdominal echography and technetium-99 m white blood cells ((99 m)TC-WBC) scan for Meckel’s diverticulum were both normal. For further investigation, the patient underwent a colonoscopy, which revealed ulcers and numerous pseudopolyps involving the ascending colon, cecum, and terminal ileum (Figs. 4, 5). During the procedure, multiple biopsies were taken and showed marked pleomorphic inflammatory infiltrates, granulation tissue, ulceration with no crypt abscesses, and very rare giant cells. The diagnosis was compatible with Crohn’s disease. For treatment in the first 3 months, we administered 5-aminosalicylic acid 50 mg/kg/day for 24 weeks, and metronidazole (20 mg/kg/day) in three divided doses for 10 weeks, then we used oral prednisone 1 mg/kg/day. During follow-up, the patient gained 0.9 kg after 6 months and Hb rose to 10 g/dl. After 18 months, the patient’s condition relapsed with bloody diarrhea, vomiting, laboratory results of Hb 6 g/dl, and body weight of 7 kg. The patient underwent a barium enema, demonstrating narrowing and strictures of the colon (Fig. 6).

Although we administered a biological treatment that included eight doses of adalimumab, our patient did not respond to treatment as abdominal pain and lower gastrointestinal bleeding remained. We planned for our patient to have a colectomy at the age of 4 years. At 1 year after surgery, the patient gained weight (2 kg) and her state is stable.

### Case 3

**Fig. 7:**
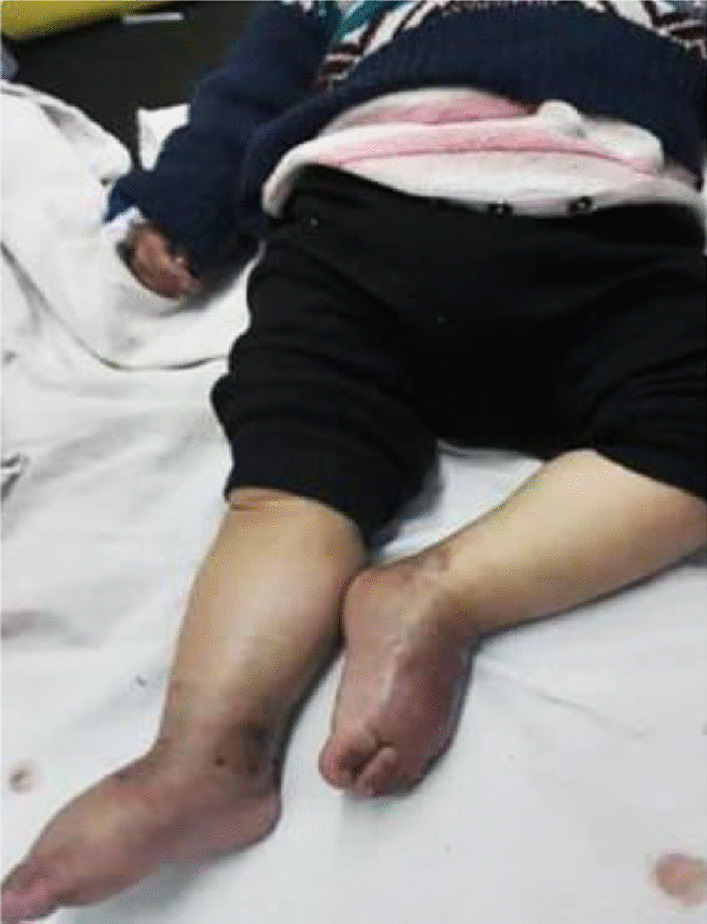
Generalized edema

**Fig. 8:**
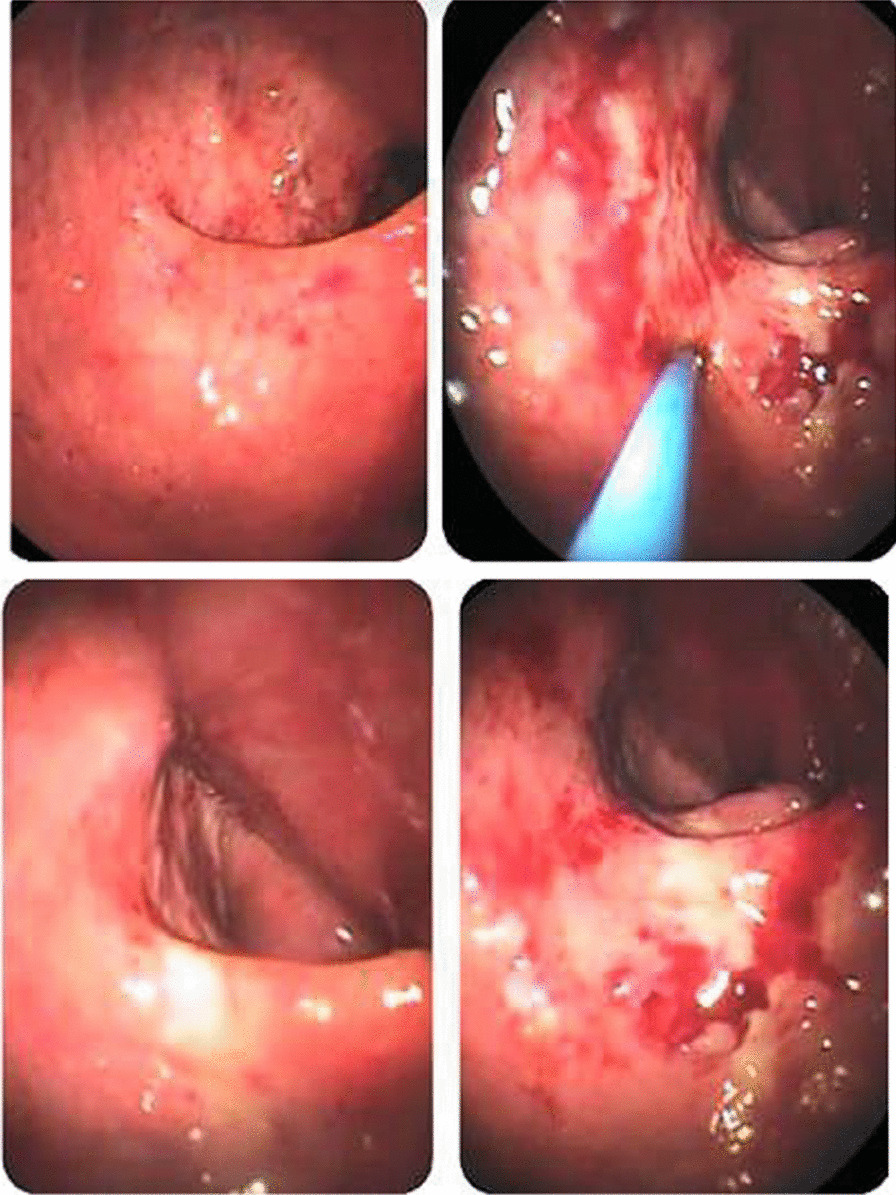
Colonoscopy showing colitis with many focal ulcerations

A 12-month-old white Syrian male baby with weight of 9.3 kg, height of 72 cm, and head circumference of 44 cm presented with 2-month history of diarrhea with slight pallor and third-degree generalized edema (Fig. 7) since the age of 1 month. Both renal and hepatic examinations were in normal ranges, as well as sweat, immunological, and septic tests. Initial laboratory results, however, showed reduction in albumin of 2.1 g/dl (nl 3.6–5.1 g/dl), total protein of 4.2 g/dl (nl 6.0–8.3 g/dl), fecal calprotectin of 180 µg/mg (nl < 50 µg/mg), and positive WBC in the stool. The esophagogastroscopy was visually normal, but the colonoscopy exhibited colitis with focal ulceration covering many spots from the rectum to ascending colon with cecal sparing (Fig. 8). Several 1-mm biopsies were taken from the ascending colon and transverse colon, besides 2-mm biopsies from the sigmoid colon and the rectum. Microscopic examination revealed an increase in the inflammatory filtrates within the lamina, containing polymorphonuclear cells invading some glands in an attempt to form diverticular abscesses consistent with ulcerative colitis. The therapy included 5-aminosalicylic acid 50 mg/kg/day for 24 weeks and metronidazole 15 mg/kg/day divided into three doses for 10 weeks. In the course of the case, follow-up for the patient was not feasible.

## Discussion

Even though the prevalence of IBD in children is expanding around the world [14, 15], it is still extremely rare, so less than 1% of the pediatric population develop neonatal or infantile IBD [16]. Between the types of IBD, ulcerative colitis appeared to be less common than Crohn’s disease in previous studies [17–20].

Although the causes of IBD remain unclear, it is probably due to a combination of hereditary and environmental factors in addition to immune dysfunction [21]. With regard to infants, IBD is frequently related to a serious course, broad colonic injury [22], and perianal lesions [20]. This is consistent with our series showing that the infants presented in cases 1 and 2 suffered from perianal lesions including ulcers and rash.

The patients presented in this series were diagnosed with Crohn’s disease in the second case, and ulcerative colitis in the first and third cases at ages 11, 7, and 12 months, respectively. All of the patients had suffered from severe diarrhea and pallor due to blood loss in feces.

The infants in case 1 and 2 were malnourished and failed to thrive. This actively demonstrates that this disease, when affecting infants, can cause weight loss and fatigue. Perianal lesions such as strictures and fistulas can be seen with the progression of the disease due to inadequate control [23], as in our first two cases. Diagnosis of IBD may be difficult, especially in infants, owing to its similar symptoms to some other more common diseases such as allergy to milk, infections, and lymphoid follicular hyperplasia [24]. This diagnosis requires several tests such as blood tests, stool studies, imaging, and endoscopy. What we noticed in case 1 was that calprotectin was significantly high and compatible with the diagnosis of inflammatory bowel disease (IBD), unlike what was shown in case 2, in which the calprotectin level was in the normal range and not compatible with the diagnosis of IBD. Upper and lower endoscopy with biopsies is considered the gold standard for IBD diagnosis [25]. This was a typical example here where colonoscopy and the pathological findings in biopsies affirmed the diagnosis of infantile IBD in these three children presented in this series.

Even after being put on conservative treatment and going through immunosuppressive therapy, the infant in case 2 required surgery with resection of the colon. A previous study showed that IBD patients with gene mutation tend to have early onset of symptoms and more resistance to medical management [26]. Our patient also showed severe and early onset of the symptoms, where he suffered from recurrent lower gastrointestinal bleeding at just 1 month of birth and was accepted into our department at the age of 7 months because of accompanying symptoms that worsened his health. Moreover, he also did not respond well to medical treatment. Shim *et al.* reported that some gene mutations are accompanied by an increased risk of failure of medical treatment and undergoing early surgical interventions. Most of the patients presented with these mutations were diagnosed with Crohn’s disease, the same as our patient presented here who needed a surgical intervention [25]. In conclusion, our patient is most likely to have a gene mutation. However, we could not relate this finding to our case because gene testing is unavailable in our country owing to financial limitations and war consequences. As for the remaining two patients, they responded well under treatment of corticosteroids, 5-acetylsalicylic acid, and antibiotics (i.e., metronidazole) and they were followed up for a few months after this treatment. However, cases 1 and 3 might show recurrence in the future, which may necessitate surgical resection of the bowel. Thus, close follow-up is needed.

Steroids were prescribed for the three cases for a short period as osteoporosis, diabetes, and disturbance in growth are consequences of long use. A previous study showed that steroid utilization is only suggested for a brief period in children [24].

Neglected IBD for a long time in addition to late diagnosis could result in severe outcomes and complications [23]. Despite the rarity of IBD in pediatric patients, early and prompt diagnosis is crucial to reduce potential severe consequences.

## Conclusion

IBDs begin rarely in infants. Approximately 25% of patients with IBDs present before the age of 20 years. Very early-onset IBD (VEO-IBD) occurs before the age of 6 years; infantile IBD occurs before the age of 2 years and is extremely rare in infants under 1 year of age. So, early diagnosis and ruling out infantile IBD despite its rarity are recommended. Over and above that, new drugs such as vedolizumab, golimumab, and less invasive treatment methods should also be taken into consideration for better response and adequate remission with improved quality of life.

## Data Availability

Not applicable.

## Abbreviations

IBDs: Inflammatory bowel diseases
CD: Crohn’s disease
UC: Ulcerative colitis
VEO-IBD: Very early-onset IBD
